# Exploring Physiological Signal Responses to Traffic-Related Stress in Simulated Driving †

**DOI:** 10.3390/s22030939

**Published:** 2022-01-26

**Authors:** Pamela Zontone, Antonio Affanni, Alessandro Piras, Roberto Rinaldo

**Affiliations:** Polytechnic Department of Engineering and Architecture, University of Udine, Via delle Scienze 206, 33100 Udine, Italy; pamela.zontone@uniud.it (P.Z.); antonio.affanni@uniud.it (A.A.); piras.alessandro@spes.uniud.it (A.P.)

**Keywords:** stress detection in drivers, electrodermal activity, electrocardiogram, motion artifact removal, Machine Learning

## Abstract

In this paper, we propose a relatively noninvasive system that can automatically assess the impact of traffic conditions on drivers. We analyze the physiological signals recorded from a set of individuals while driving in a simulated urban scenario in two different traffic scenarios, i.e., with traffic and without traffic. The experiments were carried out in a laboratory located at the University of Udine, employing a driving simulator equipped with a moving platform. We acquired two Skin Potential Response (SPR) signals from the hands of the drivers, and an electrocardiogram (ECG) signal from their chest. In the proposed scheme, the SPR signals are then processed through a Motion Artifact (MA) removal algorithm such that possible motion artifacts arising during the drive are reduced. An analysis considering the scalogram of the single cleaned SPR signal is presented. This signal, along with the ECG, is then fed to various Machine Learning (ML) algorithms. More specifically, some statistical features are extracted from each signal segment which, after being analyzed through a binary ML model, are labeled as corresponding to a stressful situation or not. Our results confirm the applicability of the proposed approach to identify stress in the two scenarios. This is also in accordance with our findings considering the SPR signal scalograms.

## 1. Introduction

Research on stress detection techniques has become more and more important in recent years. These techniques aim to automatically evaluate stress arising in individuals in relation to their health and emotional condition. In this work, we focus on the problem of automated stress detection in car drivers, since this is of paramount importance to ensure safety and wellness of professionals and people in their everyday life. As a matter of fact, the effects of emotional stress can lead to health problems [1,2] and risky behavior [3,4].

The analysis of physiological signals, as well as of physical/behavioral data, can be an effective solution for automatic stress assessment [5]. The use of Machine Learning (ML) techniques has also been proven to be effective for the classification of this kind of signals in order to automatically detect the stress level in subjects performing stress-inducing tasks.

Focusing on car driving scenarios, authors in [6,7], for instance, analyzed different physiological signals, i.e., Electrodermal Activity (EDA) [8], electrocardiogram (ECG), and respiration, to recognize stress experienced while driving. In more detail, in [6], the results of four different classifiers (i.e., support vector machines, decision trees, naive Bayes, and general Bayesian classifiers) are discussed. Only one subject was used for the experiments, and the data were collected in real driving conditions over several months. In [7], the same physiological signals are considered, but this time using 10 s windows, and the stress is still classified in two different classes. A Bayesian network was used, achieving a stress event detection accuracy equal to 82% by considering only physiological signals logged in real driving conditions. The inclusion of additional data, such as information of the current driving environment and vehicle data, as well as driving behavior history, allows the system to achieve a higher stress event detection accuracy (96%). The ground truth is determined by the drivers themselves, by stating the perceived stress level for each driving task.

ECG signals are considered in [9], where they are processed through a Support Vector Machine (SVM), a k-Nearest Neighbor (k-NN) model, and a Random Forest (RF), to evaluate how driving by following Global Positioning System (GPS) indications can affect driver’s emotions in both positive and negative ways. Another task that can modify the performance of subjects is the takeover request (TOR) in a semi-autonomous driven car. This happens in a level 3 driving automation when the driver needs to take charge of the car. Authors in [10] evaluate the effects of TOR phases by analyzing the driver’s gaze, Heart Rate (HR), Galvanic Skin Response (GSR), and facial expression. In [11], a Hidden Markov Model (HMM), which uses speed and distance between vehicles, is employed to estimate a driver’s behavior with specific induced emotions. A study that compares the car driver’s stress in a simulated autonomous and manual driving scenario, using an SVM algorithm, was introduced in [12]. The experiment was conducted using a professional driving simulator and the subjects were asked to drive both in manual and autonomous driving scenarios. The results presented demonstrate that the subjects generally appear more stressed during manual driving, proving that autonomous driving can be positively perceived by the general population. The impact of different car handling setups, e.g., understeering and oversteering, was evaluated in [13]. The goal here was to assess the driver’s response to various car settings, in order to identify a professional driver’s favorite setup or to find the most suitable setup for the majority of drivers.

Other approaches based on the use of physiological signals that do not employ ML algorithms are also proposed in literature. As a few examples, in [14], a self-similarity analysis of EDA using a wavelet-based approach is presented to evaluate the stress levels in subjects during a real-world driving experiment. In this case, only the EDA signals logged from both the foot and hand of the test subjects are considered. These signals, coming from a public dataset composed of recordings of real-world driving, are segmented into 3-minute time windows before being analyzed using self-similar processes. The authors of [15] examined ECG and eye activity, in addition to other subjective data and performance measures derived from the driving simulation, to evaluate the effects of mental workload on drivers, and also proposed a protocol to assess workload during both real and simulated tasks.

In this paper, we analyze the physiological responses of subjects as measured by EDA and ECG signals while they are driving in a simulated urban scenario. When subjects find themselves in a situation of high arousal or under stress, the stimuli generated by the Autonomic Nervous System (ANS) activity as a reaction to these situations can be evaluated through the analysis of EDA [8]. In response to the same stimuli, various parameters of the ECG signal such as Heart Rate (HR) and Heart Rate Variability (HRV) are affected. The objective of our study is to evaluate the impact of traffic conditions on drivers and develop a relatively noninvasive system that can automatically quantify the overall stress level. As a first contribution of the proposed system, we utilize the Skin Potential Response (SPR) EDA signal, and not the more commonly used Skin Conductance Response (SCR) signal. This is different from what has mostly been done in the literature, for instance in [6,7,10]. As a matter of fact, SPR appears to be less sensitive to the impedance of electrodes and to slow changes of skin impedance. SPR can be recorded easily without the need to apply current to the subjects and is commonly characterized by a quick response to stress stimuli, as opposed to SCR [8,16].

The proposed system acquires two SPR signals from the hands of a given group of subjects, as well as the ECG from their chest. Another key contribution of the proposed system is the adoption of a specific procedure to mitigate the problem of Motion Artifacts (MAs). A MA removal algorithm is applied to the SPR signals in order to remove spurious patterns due to hand movements while driving. As described below, it combines the SPR signals acquired from the two hands in order to produce a cleaned SPR signal for further processing. A set of significant features are extracted from 15 s overlapping signal blocks and used as an input to different ML classifiers, whose output is a label that discriminates between the “stress” (or “1”) and “non-stress” (or “0”) classes. The stress level over a certain time span can then be quantified by the number of “1” labels in that interval. As for the classifiers, they were trained on a large dataset of ECG and SPR signals collected in an experiment carried out in a firm specialized in car driving simulators. The trained models were then used for classification of a new collection of signals acquired from subjects during an experiment with urban simulated scenarios, characterized by the presence or absence of traffic. The new experiment involved University of Udine students and was performed on a different car simulator in our university lab; therefore, the training and test data come from different setups.

In order to better characterize the peculiar behavior of the SPR signal in this work, we also perform an offline evaluation using scalograms, which provides a time-frequency analysis of the signal [17,18]. This is an additional way to investigate the behavior of the SPR signal in the two different scenarios as a measure of the emotional responses of the subjects while driving. The computation of the SPR scalograms requires knowledge of the entire SPR signal logged from the subjects for each scenario and it is not computed in real time. On the other hand, the application of the ML classifiers is carried out in each 15 s interval, with a new interval chosen every 5 s. The selected features from both SPR and ECG signals contain extensive information about the signals, and are extracted from each 15 s interval. In this way, the proposed classifiers can operate in real time, on the basis of more complete information than that provided by the scalogram.

Here, we extend the work presented in [19], where we considered only the EDA signal for classification, and only the results obtained using an SVM classifier were discussed. Moreover, our work extends the results of [12,13], where we used a similar approach, but in different setups and scenarios (as introduced before) compared to this study. In summary, our work proposes a complete system to evaluate traffic-related stress in drivers, including the sensor design for SPR signals, the MA removal algorithm, and the setup of all the parameters of the ML architecture. Our results suggest that the proposed system, which can be implemented in real time and with limited detection delay, is effective in discriminating the effect of traffic on different subjects.

The paper is structured as follows. The next section introduces the fundamental blocks of the proposed scheme. We describe the sensor we used for the acquisition of both the SPR and ECG signals. We then examine the MA removal algorithm that allows us to reduce possible motion artifacts arising in the SPR signals acquisition (Section 2.1), and the ML algorithms used for stress classification (Section 2.3). The experimental setup is described in Section 2.4. For signal analysis and interpretation of the SPR signal, we propose the use of scalograms, with an overview of the scalogram representation provided in Section 2.2. Section 3 discusses the experimental results of our study, first considering the SPR scalograms and then considering both the EDA and ECG signals as a measure of the subjects’ stress response. The results in terms of positive intervals (with stress) obtained when considering the different ML models are included in this section. At the end, some conclusions are drawn (see Section 4).

## 2. Materials and Methods

**Figure 1:**
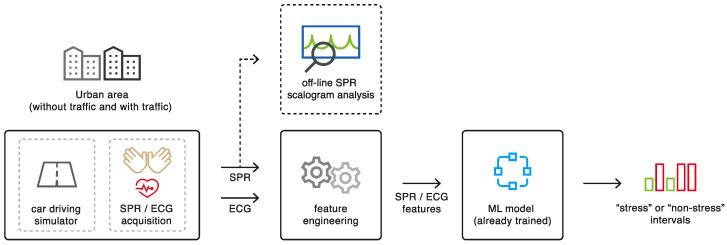
Block scheme of the proposed system.

In this section we will examine the fundamental blocks of the proposed system (Figure 1) and present our experimental setup. As detailed below, subjects were asked to drive in a simulated urban environment in two different situations: with traffic and without traffic. The ECG and SPR signals were recorded and processed to calculate a set of features for each time interval. Finally, the feature vectors were classified into the “stress” and “non-stress” classes based on ML techniques trained on a larger dataset. Each block of Figure 1 will be better explained below. The scheme of Figure 1 includes an off-line block not operating in real time, which processes the entire cleaned SPR waveform by means of the scalogram representation, which we will describe in Section 2.2.

### 2.1. The Sensor and the MA Removal Algorithm

**Figure 2:**
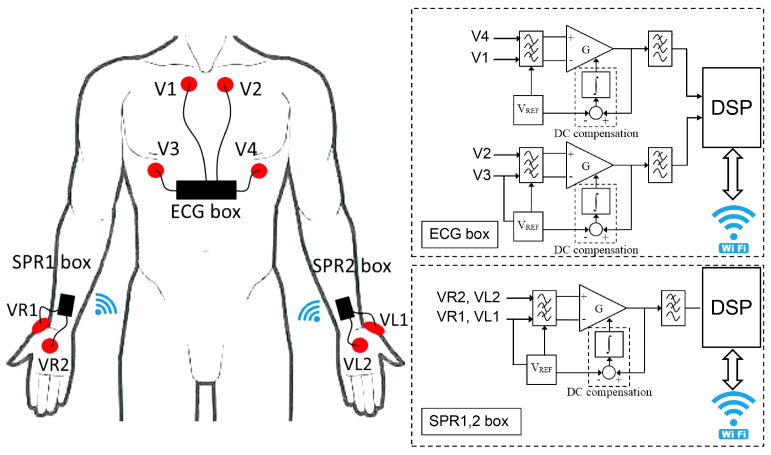
Electrode positioning and sensor block diagram.

The sensor system used for data acquisition was developed by the authors and has been described in detail and characterized in [20]. The system is composed of an ECG sensor and two SPR sensors that are battery operated and connected through WiFi, forming a body wireless network (see Figure 2). The two SPR sensors (worn on the wrists like smartwatches) acquire the SPR signal from each hand and transmit data (via UDP protocol) to the ECG sensor, which acts as an access point for the body network. The ECG sensor, worn on the chest, acquires two ECG derivations and manages the connection with the SPR sensors and a laptop for data transmission. Each of the three sensors is composed of an analog front end, a DSP for signal conversion and transmission, and a WiFi module for wireless connection. The analog front end of each sensor has an input impedance of 100 MΩ and conditions the low-level signals on the skin into high-level signals suitable for A/D conversion.

In detail, the SPR front end is a band-pass differential amplifier in the range [0.08, 8] Hz with gain 160; thus, the full-scale (FS) input range for the SPR signal is ±10 mV. The accuracy of the SPR reading is 0.15% FS (corresponding to 30 μV) and the resolution is 4.9 μV. The double channel ECG analog front end is a couple of band-pass differential amplifiers in the range [0.08, 75] Hz with gain 320; thus, the input range for the ECG signals is ±5 mV. The accuracy of the ECG reading is 0.05% FS (corresponding to 5 μV) and the resolution is 2.4 μV. The analog signals are converted using 12-bit A/D converters on board the DSPs, which acquire the signals at a rate of 200 Sa/s.

Once the time-aligned data are transmitted to the laptop, the motion artifact (MA) removal algorithm combines the two SPR signals in order to remove the artifacts due to hand action on the steering wheel. The two SPR signals acquired from the hands of the subjects are converted into a single signal that better represents the activity of the ANS. The MA algorithm is based on the assumption that the local energy of the raw SPR signal is the sum of two contributions; one (desired) is the ANS activity that is the same on both hands, the other (undesired) is due to the motion of the hands on the steering wheel, increasing the local energy of the signal. Assuming that the motion artifact rarely appears simultaneously on both hands, the output signal of the MA removal block is thus obtained as a weighted average of the two raw SPR signals, giving heavier weight to the less perturbed signal, which is the one with the lowest local energy. The algorithm is described in detail in [21].

### 2.2. SPR Scalograms

We propose the use of scalograms for SPR signal analysis and interpretation. A scalogram is a graphical representation of the absolute value of the Continuous Wavelet Transform (CWT) of a signal that provides a time–frequency signal description [17,18]. Scalogram plots are commonly used in order to localize signal behavior in time and frequency, since they actually provide an instantaneous signal frequency estimation. They are also useful for analyzing localized signal discontinuities, which are typical of SPR responses to stress-evoking events [8]. CWT is calculated from possibly complex-valued time/frequency localized wavelets, which are derived from one single function ψ(t) (the mother wavelet) through time shifting and scaling, namely,

$$\psi_{ab}\left(t\right)=\frac{1}{\sqrt{a}}\;\psi \left(\frac{t-b}{a}\right).$$

Here, a>0 represents the scale factor and *b* is the shift factor. The CWT of a signal x(t) is computed as

$$CWT\left[x\left(t\right)\right]=W_{\psi }\left(a,b\right)=\frac{1}{\sqrt{a}}\int_{-\infty }^{-\infty }x\left(t\right)\;\psi^{*}\left(\frac{t-b}{a}\right)dt$$

i.e., as the inner product between x(t) and the scaled and shifted version of the mother wavelet. Assuming that the mother wavelet satisfies the appropriate hypotheses, the CWT therefore provides an overcomplete representation of the signal, where the scale *a* determines the bandwidth and *b* determines the time position of the time-frequency window. Various wavelet functions can be utilized for the CWT. In our work, we use the generalized Morse analytic wavelet, which in the frequency domain can be written as [22]

$$\Psi_{\beta ,\gamma }\left(\omega \right)=\int_{-\infty }^{-\infty }\psi_{\beta ,\gamma }\left(t\right)\;e^{-j\omega t}dt=U\left(\omega \right)\;k_{\beta ,\gamma }\;\omega^{\beta }\;e^{-\omega^{\gamma }}.$$

In this equation, $k_{\beta ,\gamma }$ is a normalization constant, U(ω) denotes the unit step function, and γ and β are parameters that define the exact wavelet shape [22]. In our experiments, we set γ=3 and the time–bandwidth product β·γ=60. Efficient algorithms for the computation of Morse wavelets are available [23]. We use the Matlab implementation of the corresponding scalograms. As we will see in Section 3.1, we evaluate the SPR scalograms to assess if changes in the subjects’ emotional responses can also be revealed with this particular method.

### 2.3. Feature Extraction and ML Classification

The last two blocks of the proposed system in Figure 1 refer to the extraction of the features and their subsequent classification with ML techniques. To evaluate which of the two scenarios was more stressful for each subject, we utilize various binary ML classifiers that were previously trained on a larger dataset.

The dataset was obtained in a previous experiment [21] that was performed in a leading company in the field of professional car simulators (VI-grade s.r.l., Tavagnacco, Italy [24]). We monitored the emotional responses of 18 drivers to different stress-inducing events by acquiring their SPR signals (again, from both hands) and their ECG signals during a simulated drive. The subjects had to manually drive along a 67 km highway, facing 12 obstacles meant to evoke stress placed in predetermined points along the track. These 12 obstacles were: double lane change (right to left or left to right), tire labyrinth, sponsor block (from left or from right), slalom (from left or from right), lateral wind (from left or from right), jersey LR, tire trap, and stop. After normalization, the cleaned SPR and ECG signals were divided into 15 s blocks with 10 s overlap. We extracted eight features from each block. For the SPR signal, the features were the block variance, the energy, the mean absolute value, the mean absolute derivative, and the maximum absolute derivative. For the ECG, we computed the mean value of normal-to-normal R-peak time difference in consecutive QRS ECG complexes (RR, also known as NN), the standard deviation of RR intervals (SDNN), and the mean value of the Heart Rate (HR). These features were grouped together to create the feature vectors to be sent to various ML algorithms. Each feature was also rescaled through a min-max normalization, so that in the end they ranged from 0 to 1. Due to the 10 s overlap between consecutive blocks, a feature vector was computed every 5 s. To provide the ground truth to train the ML models, we assumed that the signal blocks within or intersecting the time intervals where obstacles were encountered belonged to the class “stress”, and all the others to the class “non-stress”. In total, the training dataset was composed of 3195 intervals for each class. After the test dataset was classified, we also applied a relabel procedure in order to lessen the number of isolated labels [21].

We examined the results of an SVM classifier, an RF classifier, and a Decision Tree (DT) classifier, which resulted in similar accuracies. In Table 1, we show the performance of these classifiers, also including the sensitivity, specificity, Balanced Accuracy (BA), and Geometric Mean (GM). These two last parameters were obtained by computing the arithmetic mean of sensitivity and specificity, and the geometric mean of sensitivity and specificity, respectively. We use the “leave-one-person-out” procedure; i.e., for the training process, we use the data of all subjects, leaving one subject out, which is the one on which the classifier is tested. The values in Table 1 are the average of the results considering all of the subjects. All of these ML classifiers were implemented in Matlab, with the help of the Machine Learning Toolbox. A 10-fold cross validation step was also applied to all of these algorithms for hyperparameter optimization (see also [25]).

The ML classifiers, after being trained on the described larger dataset, were used for the testing procedure on the dataset composed of the SPR and ECG acquired from the subjects while they were driving in a simulated urban area. The results will be discussed in Section 3.2.

### 2.4. Experimental Setup

As mentioned before, for the experiment described in this work, we use a car driving simulator located in the BioSens Lab [26] at the University of Udine. It consists of a moving platform (namely, the DOF Reality Professional P2) that allows movement along two axes, i.e., pitch and roll. A steering wheel (Logitech G29), pedals, gearbox, and seat are firmly fixed on the platform and a curved screen completes the setup for the manual driving experience simulation. The virtual environment and traffic are simulated by the City Car Driving simulation software [27]. We tested ten students of the University of Udine in the 23–29 age range with at least three years of driving experience. The subjects tested filled out an informed consent module before using the simulator. The principles of the Declaration of Helsinki were also respected during the tests [28]. All of the 10 subjects were asked to use the simulator and to drive without any constraints along a predetermined track in an urban area. The City Car Driving software allows the subjects to be immersed in a simulated urban area and provides credible traffic conditions. While driving, the subjects encounter unpredictable events that can cause a stress reaction, such as pedestrians invading the road and cars behaving erratically, occupying the other lane, or reducing speed without notice.

**Figure 3:**
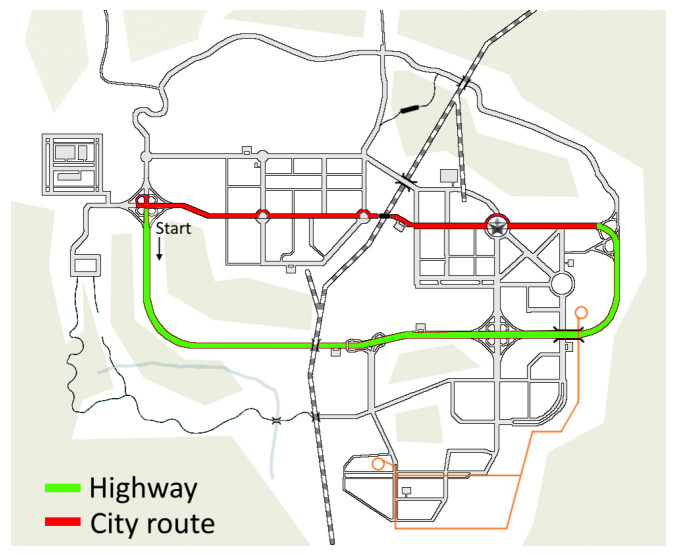
Map of the course, which consists of a highway section and a city route.

We were able to place these stressors in the simulation in a way that they happen with a typology and a frequency that are comparable for each run, even if they are not exactly the same in a deterministic way. The whole track is displayed in Figure 3. The green lane shows the highway and the red lane delineates the city route. The subjects were asked to drive immersed in two scenarios: one is identified by the elimination of cars and people, whereas the other one is identified by their presence. In the simulation with traffic, the traffic density is low, but the attitude of cars and people is put in the “very aggressive” setting, which implies a more impulsive and unpredictable temperament of cars and people, with more frequent events of cars invading the subjects’ lane or people jaywalking into the streets. The traffic-free scenario was tested by half of the subjects before the traffic scenario (Subjects 1, 2, 3, 4, and 10), while the other half drove the traffic scenario first and the traffic-free one later (Subjects 5, 6, 7, 8, and 9). The track depicted in Figure 3 let us record roughly ten minutes of data for each simulation, nearly evenly divided between the city route and highway sections.

## 3. Results and Discussion

In this section, we will discuss the experimental results obtained considering the scalogram of the SPR signals. As mentioned, this analysis provides an overall off-line evaluation, which shows the signal differences in the two driving scenarios. We will then present the results considering both the SPR and ECG signals logged from the subjects during the course in the two different urban scenarios (with and without traffic), comparing the classification performance obtained with the various ML algorithms.

### 3.1. SPR Scalogram Analysis

A first assessment considering only the SPR signal was carried out to evaluate if the physiological responses of the subjects to traffic situations can be analyzed through the scalogram. In our work, we analyzed the scalograms of the cleaned SPR signals of the ten subjects in both the traffic and non-traffic scenarios. For each subject, the signals were normalized by subtracting the mean and dividing the result by the standard deviation of the concatenation of the signals obtained in the two driving scenarios, for that subject. This way, we can fairly compare the different subjects in their response to the test, as well as the response of each driver to the two scenarios.

To compute the scalogram with the Matlab routines, we set the voices per octave, which is a parameter used to discretize the CWT scale values, to 12. We also specified the sampling frequency of the signal so that a scale-to-frequency conversion is carried out, giving back the frequencies measured in Hz (actually, these are pseudo-frequencies associated to the scale values, since there is not an exact relationship between scale and frequency). One way to do this is by determining, for each value of the scale *a*, the center frequency of the wavelet in Hz, identifying its peak value in the frequency domain.

**Figure 4:**
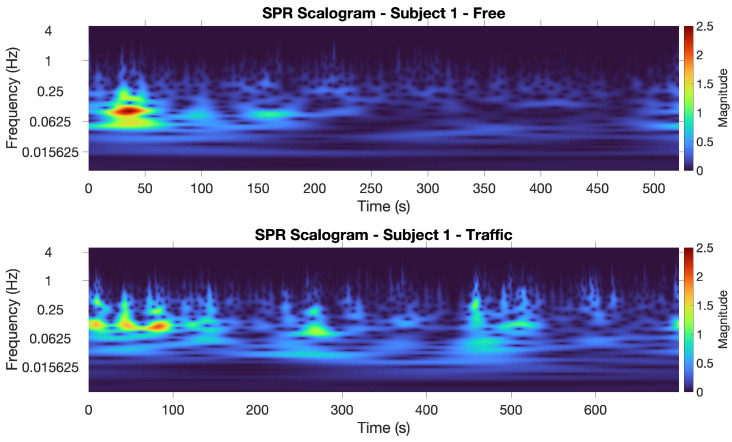
SPR scalograms for Subject 1: non-traffic scenario (**top**) and traffic scenario (**bottom**).

Figure 4 displays the SPR scalograms obtained for Subject 1. We show the scalogram in the non-traffic scenario at the top, and the traffic one at the bottom. The presence of several high-magnitude episodes in the traffic scenario is clearly evident, as opposed to the non-traffic scenario, where a few low-magnitude episodes occur (besides one single high-magnitude episode, at the beginning of the experiment, that could be due to a temporary state of excitement for taking the test).

**Figure 5:**
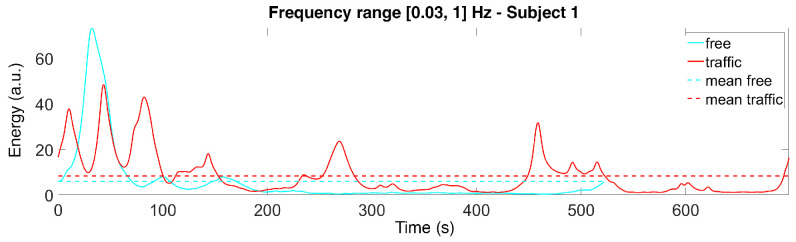
Signals obtained as a sum of the squared absolute values of the CWT coefficients in the selected frequency range for Subject 1 (in both scenarios).

**Figure 6:**
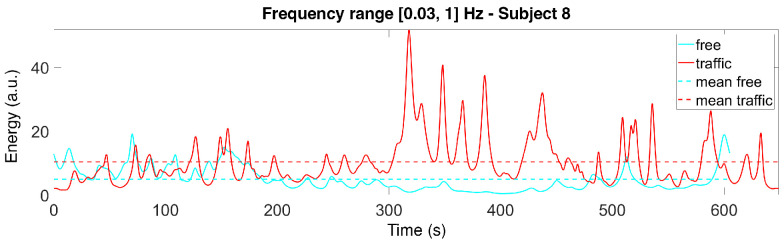
Signals obtained as a sum of the squared absolute values of the CWT coefficients in the selected frequency range for Subject 8 (in both scenarios).

Considering all of the subjects’ scalograms, we notice that the maximum magnitudes of the CWT coefficients almost always appear in the [0.03, 1] Hz range. Therefore, for each subject and each time instance, we compute the sum of the squared absolute values (i.e., the energy) of the CWT coefficients in this frequency range. The results are plotted in Figure 5 and Figure 6 for Subjects 1 and 8, showing the energy computed along the track.

For each subject, we then calculate the mean of the energy for the entire duration of the experiment, in the two different situations. The results are reported in Table 2.

**Figure 7:**
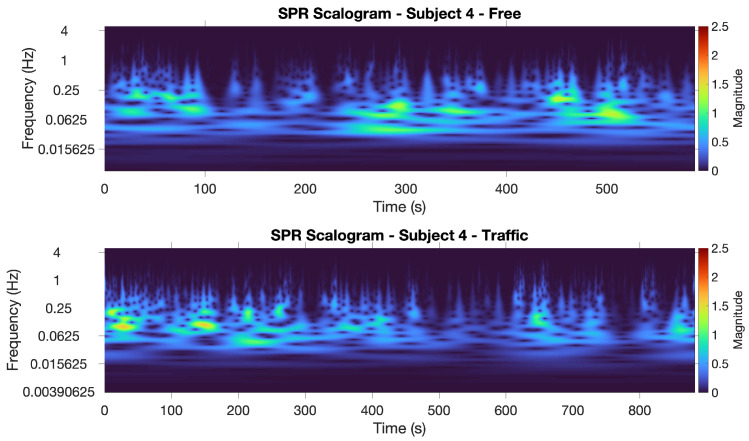
SPR scalograms for Subject 4: non-traffic scenario (**top**) and traffic scenario (**bottom**).

The differences between these values are also included in the table. We can see that, considering the mean value of the energy of the CWT coefficients, two subjects out of ten exhibited a higher energy in the traffic-free situation. The scalograms for one of these two subjects, i.e., Subject 4, are shown in Figure 7. In this case, the large number of high-magnitude episodes appearing in the non-traffic scenario opposed to the traffic scenario is clearly noticeable. Our evaluation of the SPR scalograms suggests that these two subjects appear to experience more stress in the non-traffic scenario and this is confirmed by the results obtained with the ML classifiers using both the SPR and ECG signals (see the next section). We can also note that Subjects 3 and 4 drove in the non-traffic scenario first, and this may have influenced their emotional responses.

### 3.2. ML Classification Results

As shown in Figure 1, the cleaned SPR signal (after the MA removal block) and the ECG signal were then processed to calculate the feature vectors for the classifiers. Regarding the ECG, we extract the R peak locations through the Pan–Tompkins algorithm [29] and we correct the ectopic beats as in [30]. We then derive the RR signal with equidistant sampling by interpolating the non-equidistantly sampled RR interval time series (through a cubic spline interpolation). In the end, we normalize all of the RR signals to make them comparable among the different subjects (using the same procedure described for the SPR signal).

The SPR and RR signals are then sent to the three binary ML classification algorithms presented previously, which are now only used for testing. Considering each subject driving in the two situations (with and without traffic), for each 15 s interval, we are able to compute the eight features described in Section 2.3. As already mentioned, a new interval is picked 5 s after the previous one, meaning that there is a 10 s overlap between successive intervals. The SVM, RF, and DT classifiers, by analyzing these features, output a label for each 15 s interval that indicates whether the interval is classified in the “stress” or “non-stress” class. In this way, we are able to calculate for each subject and each driving situation the final number of labels equal to “1” or “0”, i.e., the total number of intervals that the classifiers identify as “stress” or “non-stress”. Table 3 displays the percentage of the intervals marked as “1” (or “stress” intervals) observed for each classifier and for each subject, taking into account the complete track in the two different situations.

**Figure 8:**
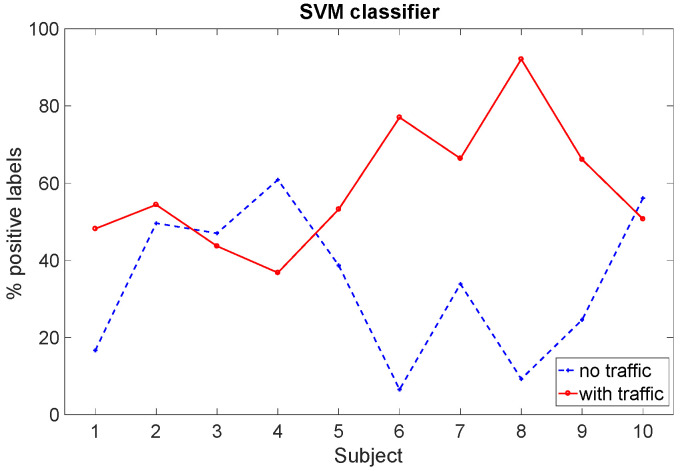
Total number of intervals identified as “stress” by the SVM classifier (in %) for each subject in the two different driving situations.

**Figure 9:**
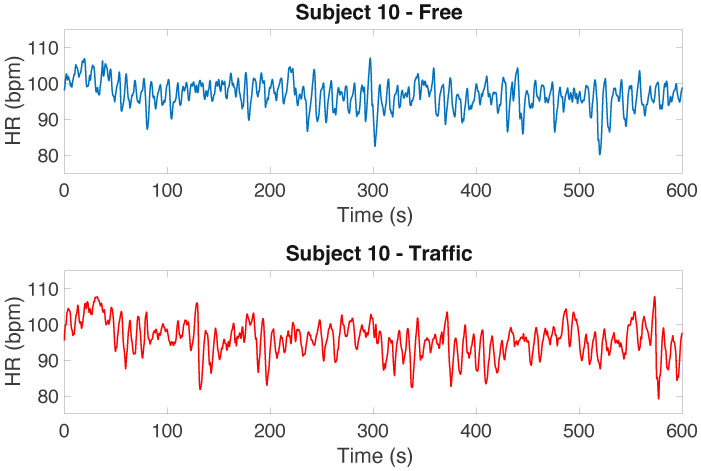
HR signal for Subject 10 in the two scenarios: without traffic (**top**) and with traffic (**bottom**).

As an example, Figure 8 graphically depicts the values indicated in Table 3 for the SVM classifier. Looking at this figure, but also considering the data reported in Table 3, we can observe that the driving situation with traffic seems to generate more stress when compared to the driving situation without traffic for the majority of the subjects. In particular, according to the results obtained with the SVM classifier, three subjects out of ten (i.e., Subjects 3, 4, and 10) seem to experience more stress when dealing with the non-traffic scenario. Note that two of them are the same ones that appear to be more stressed in the non-traffic scenario by analyzing their scalogram, and that the difference between the two scenarios for Subject 10 is very small. For the RF classifier, two subjects (i.e., Subjects 4 and 10) appear to be more stressed in the non-traffic scenario, whereas for the DT classifier, four subjects (i.e., Subjects 2, 3, 4, and 10) appear to be more stressed in the non-traffic scenario (even if the difference between the percentage of the positive labels in the traffic scenario and the ones in the non-traffic scenario for Subjects 2 and 3 is again very small). Note that the order in which the subjects conducted the experiment (starting with the non-traffic situation and continuing with the traffic situation, or vice versa) could also have influenced the results in terms of the physiological reaction arising in the subjects. Comparing these results to those presented in [19], where only the SPR signal was considered, we observe that the inclusion of the ECG features may not have significantly affected the classification procedure. As an example, Figure 9 shows the HR for Subject 10, with no clear difference between the signal characteristics in the two scenarios. In an urban scenario more effort is needed to drive, so we believe that this subject’s physical activity could have masked the variability of ECG associated with stress episodes, making them less detectable. For the same reason, we did not include the root mean square of the subsequent RR interval differences (RMSSD) feature, which is often used for classification [21]. RMSSD is in fact useful to discriminate episodes corresponding to increasing or decreasing HR, which we did not observe in the experiment.

Even if the scope of this work is to compare the emotional responses of the subjects while driving in the entire track with and without traffic, in order to have an even more complete overview of the subjects’ stress reactions, we examined the highway and city route sections separately. We report in Table 4 the total number of intervals marked as “stress” by the SVM classifier (in %) for each subject and each road section. We can note that, on average, the highway section appears to be more stressful than the city route section, in particular in the traffic scenario.

Considering again the course as a whole (highway and city route together), a further analysis was carried out considering a statistical non-parametric test (Wilcoxon signed rank test) comparing the data for the ten subjects in the two situations, traffic and non-traffic. By using the ten values representing the positive label percentage of the subjects in the traffic scenario and the ten values representing the positive labels of the subjects in the non-traffic scenario as input, this statistical test provides a *p*-value equal to p=0.017 considering the SVM classifier, p=0.026 for the RF classifier, and p=0.038 for the DT classifier. This confirms that, for each classifier, there is a significant difference between the number of positive labels collected in the two scenarios or, in other words, the two scenarios do cause a different emotional response in the test subjects.

**Figure 10:**
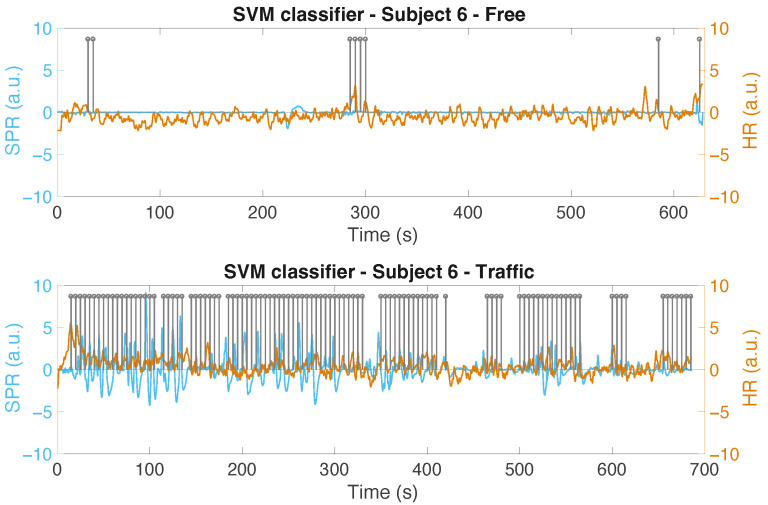
The output of the SVM classifier for Subject 6 in both scenarios: without traffic (**top**) and with traffic (**bottom**).

**Figure 11:**
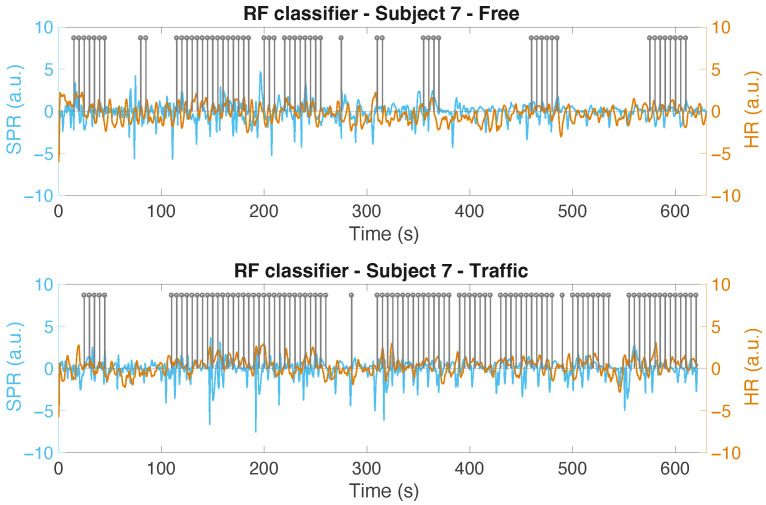
The output of the RF classifier for Subject 7 in both scenarios: without traffic (**top**) and with traffic (**bottom**).

**Figure 12:**
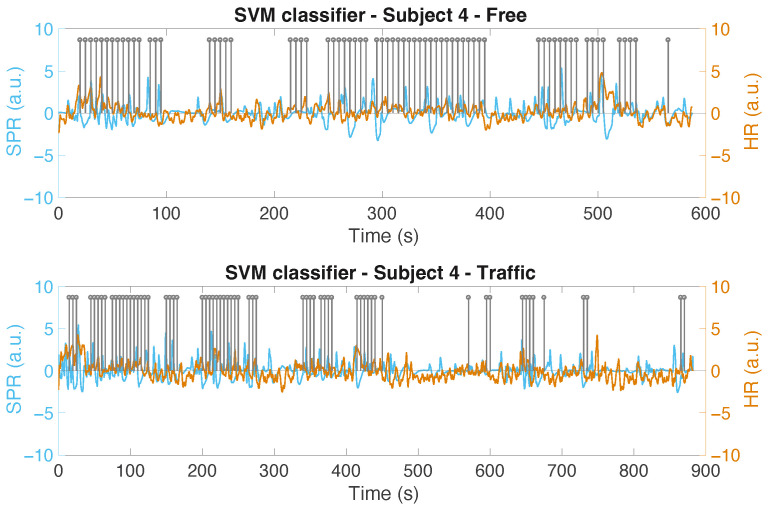
The output of the SVM classifier for Subject 4 in both scenarios: without traffic (**top**) and with traffic (**bottom**).

In Figure 10, we show the output of the SVM for Subject 6 (in the two scenarios), where the difference between the positive labels in the traffic and non-traffic situation is among the highest positive ones we obtain. We only show the positive labels (denoted as “1”) using a grey stem, located at the end of the corresponding 15 s classified interval. For simplicity’s sake, the labels of the intervals classified as “non-stress” are not displayed. The cleaned and normalized SPR signals, along with the normalized HR of the subject in the two scenarios, are also shown in the figure. Another example, using the RF classifier is shown in Figure 11 for Subject 7. In Figure 12, the output of the SVM classifier for Subject 4 is depicted. This is the case where the difference between the positive labels in the traffic and non-traffic scenario is the greatest, in negative terms, for all the classifiers. Looking at these figures, however, we can see that the classifiers, by analyzing the SPR and ECG signals, are able to properly detect the stress episodes along the course in an urban area.

## 4. Conclusions

In this paper, we described a system that allows us to analyze the stress reactions of subjects while driving on a simulator with a motorized platform in two different urban scenarios, designed with the simulation software City Car Driving. We consider the SPR logged from the subjects’ hands and the ECG logged from their chest. A first analysis of the SPR scalograms was carried out. For each subject, we evaluated the energy of the CWT coefficients in a given range of frequencies (corresponding to the highest magnitude coefficients). Considering the mean of the energy computed on the entire track, we note that two subjects out of ten seemed to be more stressed during the driving scenario without traffic. This was also confirmed by an additional analysis performed using both the SPR and ECG signals processed through various ML classification algorithms. By counting the number of output labels equal to “1”, which identify the stress intervals, and the output labels equal to “0”, which identify the non-stress intervals, we can evaluate how the subjects responded to the different scenarios. Our findings show that the majority of subjects appear to have been more stressed during the driving scenario characterized by the presence of traffic. More specifically, considering the SVM, seven subjects out of ten appeared to have been more stressed in the traffic scenario. With the RF classifier, eight subjects out of ten appear to have been more stressed in the traffic scenario, whereas with the DT classifier, four subjects out of ten appear to have been more stressed in the non-traffic scenario (even if the difference between the percentage of positive labels is very small in two cases). A significant statistical difference between the number of positive labels generated in the two scenarios for each subject is also confirmed by the Wilcoxon test results. In the end, we can see that our system performs well in recognizing stress episodes by using SPR and ECG signals. Increasing the number of subjects involved and analyzing other and new physiological signals in addition to SPR and ECG are possible paths for further extension of this research activity.

## Figures and Tables

**Table 1:** SVM, RF, and DT classification performance.

	Accuracy (%)	Sensitivity (%)	Specificity (%)	BA (%)	GM (%)
SVM	79.89	68.98	87.31	78.14	77.49
RF	79.42	70.06	85.80	77.93	77.43
DT	79.51	70.96	85.40	78.18	77.72

**Table 2:** Mean energy in the selected frequency range, computed considering the entire duration of the experiment (for all of the subjects). The numeric difference between them (with traffic–no traffic) is also reported.

Mean Energy (a.u.)										
Subject	1	2	3	4	5	6	7	8	9	10
Traffic	8.27	8.22	6.61	6.99	14.96	14.96	7.67	10.33	11.51	9.68
No Traffic	5.85	6.80	9.09	8.77	0.58	0.23	7.22	4.92	3.43	5.30
Traffic − No traffic	2.42	1.42	−2.48	−1.78	14.38	14.72	0.44	5.41	8.07	4.38

**Table 3:** Total number of intervals identified as “stress” (in %) for each classifier and each subject in the two driving situations. The numeric difference between them (with traffic–no traffic) is also displayed.

**SVM**											
Subject	1	2	3	4	5	6	7	8	9	10	Mean (%)
% “1” Traffic	48.18	54.40	43.67	36.78	53.23	77.04	66.39	92.13	66.12	50.71	58.86
% “1” No traff.	16.67	49.62	47.01	60.87	38.66	6.50	33.87	9.24	24.58	56.10	34.31
% “1” Diff.	31.51	4.78	−3.34	−24.09	14.57	70.53	32.52	82.88	41.54	−5.38	24.55
**RF**											
Subject	1	2	3	4	5	6	7	8	9	10	Mean (%)
% “1” Traffic	54.74	58.40	48.10	44.83	55.65	74.81	76.23	92.13	71.07	52.86	62.88
% “1” No traff.	24.51	56.49	47.01	66.96	25.21	2.44	45.16	19.33	31.36	73.17	39.16
% “1” Diff.	30.23	1.91	1.09	−22.13	30.44	72.38	31.07	72.80	39.72	−20.31	23.72
**DT**											
Subject	1	2	3	4	5	6	7	8	9	10	Mean (%)
% “1” Traffic	51.09	52.00	46.84	41.38	62.90	77.04	69.67	85.83	60.33	45.00	59.20
% “1” No traff.	27.45	52.67	47.01	63.48	46.22	10.57	33.06	15.97	37.29	59.35	39.31
% “1” Diff.	23.64	−0.67	−0.17	−22.10	16.68	66.47	36.61	69.86	23.04	−14.35	19.90

**Table 4:** Total number of intervals identified as “stress” (in %), considering the SVM classifier for each subject and each road segment (in the two driving situations). The numeric difference between them (with traffic–no traffic) is also displayed.

**SVM-Highway**											
Subject	1	2	3	4	5	6	7	8	9	10	Mean (%)
% “1” Traffic	59.38	60.00	43.55	59.30	61.40	95.00	57.14	88.33	67.86	75.00	66.70
% “1” No traff.	34.78	68.33	57.69	54.55	24.00	3.85	53.33	15.38	14.00	80.00	40.59
% “1” Diff.	24.59	−8.33	−14.14	4.76	37.40	91.15	3.81	72.95	53.86	−5.00	26.10
**SVM-City Route**											
Subject	1	2	3	4	5	6	7	8	9	10	Mean (%)
% “1” Traffic	38.36	49.23	43.75	14.77	46.27	62.67	74.24	95.52	64.62	34.52	52.39
% “1” No traff.	1.79	33.80	38.46	64.79	49.28	8.45	15.63	4.48	32.35	39.73	28.87
% “1” Diff.	36.57	15.43	5.29	−50.02	−3.01	54.22	58.62	91.04	32.26	−5.20	23.52

## Data Availability

The data presented in this study are available on request from the corresponding author.

## Abbreviations

The following abbreviations are used in this manuscript:

SPR	Skin Potential Response
EDA	Electrodermal Activity
ECG	Electrocardiogram
MA	Motion Artifact
ML	Machine Learning
SVM	Support Vector Machine
RF	Random Forest
DT	Decision Tree

## References

[1] Amichai-Hamburger Y. (2009). Technology and Psychological Well-Being.

[2] Härmä M., Viikari-Juntura E., O’Donoghue-Lindy L. (2015). Scandinavian Journal of Work, Environment & Health: 40 years of innovative research with societal impact in the field of occupational health. Scand. J. Work. Environ. Health.

[3] Scott-Parker B., Jones C.M., Rune K., Tucker J. (2018). A qualitative exploration of driving stress and driving discourtesy. Accid. Anal. Prev..

[4] Huang Y.W., Lin P.C., Wang J. (2018). The influence of bus and taxi drivers’ public self-consciousness and social anxiety on aberrant driving behaviors. Accid. Anal. Prev..

[5] Greene S., Thapliyal H., Caban-Holt A. (2016). A survey of affective computing for stress detection: Evaluating technologies in stress detection for better health. IEEE Consum. Electron. Mag..

[6] Rigas G., Goletsis Y., Bougia P., Fotiadis D.I. (2011). Towards driver’s state recognition on real driving conditions. Int. J. Veh. Technol..

[7] Rigas G., Goletsis Y., Fotiadis D.I. (2012). Real-time driver’s stress event detection. IEEE Trans. Intell. Transp. Syst..

[8] Boucsein W. (2012). Electrodermal Activity.

[9] Li J., Lv J., Oh B.S., Lin Z., Yu Y.J. (2020). Identification of Stress State for Drivers Under Different GPS Navigation Modes. IEEE Access.

[10] Du N., Yang X.J., Zhou F. (2020). Psychophysiological responses to takeover requests in conditionally automated driving. Accid. Anal. Prev..

[11] Liu Y., Wang X. (2020). Differences in Driving Intention Transitions Caused by Driver’s Emotion Evolutions. Int. J. Environ. Res. Public Health.

[12] Zontone P., Affanni A., Bernardini R., Del Linz L., Piras A., Rinaldo R. (2020). Stress Evaluation in Simulated Autonomous and Manual Driving through the Analysis of Skin Potential Response and Electrocardiogram Signals. Sensors.

[13] Zontone P., Affanni A., Bernardini R., Del Linz L., Piras A., Rinaldo R. Emotional response analysis using electrodermal activity, electrocardiogram and eye tracking signals in drivers with various car setups. Proceedings of the 2020 28th European Signal Processing Conference (EUSIPCO).

[14] El Haouij N., Ghozi R., Poggi J.M., Sevestre-Ghalila S., Jaïdane M. (2019). Self-similarity analysis of vehicle driver’s electrodermal activity. Qual. Reliab. Eng. Int..

[15] Peruzzini M., Tonietti M., Iani C. (2019). Transdisciplinary design approach based on driver’s workload monitoring. J. Ind. Inf. Integr..

[16] Bari D., Yacoob H., Tronstad C., Kalvøy H., Martinsen Ø (2018). Electrodermal responses to discrete stimuli measured by skin conductance, skin potential, and skin susceptance. Skin Res. Technol..

[17] Vetterli M., Kovačevic J. (1995). Wavelets and Subband Coding.

[18] Sejdic E., Djurovic I., Stankovic L. (2008). Quantitative Performance Analysis of Scalogram as Instantaneous Frequency Estimator. IEEE Trans. Signal Process..

[19] Zontone P., Affanni A., Piras A., Rinaldo R. Stress recognition in a simulated city environment using Skin Potential Response (SPR) signals. Proceedings of the 2021 IEEE International Workshop on Metrology for Automotive (MetroAutomotive).

[20] Affanni A. (2020). Wireless sensors system for stress detection by means of ECG and EDA acquisition. Sensors.

[21] Zontone P., Affanni A., Bernardini R., Piras A., Rinaldo R., Formaggia F., Minen D., Minen M., Savorgnan C. (2020). Car driver’s sympathetic reaction detection through electrodermal activity and electrocardiogram measurements. IEEE Trans. Biomed. Eng..

[22] Lilly J.M., Olhede S.C. (2012). Generalized Morse Wavelets as a Superfamily of Analytic Wavelets. IEEE Trans. Signal Process..

[23] (2016). jLab: A Data Analysis Package for Matlab. http://www.jmlilly.net/jmlsoft.html.

[24] VI-Grade Srl. https://www.vi-grade.com.

[25] Zontone P., Affanni A., Bernardini R., Linz L.D., Piras A., Rinaldo R. (2020). Supervised learning techniques for stress detection in car drivers. Adv. Sci. Technol. Eng. Syst. J..

[26] BioSens Lab. http://phd.diegm.uniud.it/senesors-and-biosignals-lab.

[27] City Car Driving Simulator, PC Game. https://citycardriving.com.

[28] Association W.M. (2013). World Medical Association Declaration of Helsinki: Ethical Principles for Medical Research Involving Human Subjects. JAMA.

[29] Pan J., Tompkins W.J. (1985). A Real-Time QRS Detection Algorithm. Biomed. Eng. IEEE Trans..

[30] Tarvainen M.P., Niskanen J.P., Lipponen J.A., Ranta-Aho P.O., Karjalainen P.A. (2014). Kubios HRV—Heart rate variability analysis software. Comput. Methods Programs Biomed..

